# Clinical Characteristics and In-Hospital Outcomes in Dialysis Patients with Septic Arthritis

**DOI:** 10.3390/medicina58030401

**Published:** 2022-03-07

**Authors:** Hsin-Tzu Yeh, Shuh-Kuan Liau, Kuang-Yu Niu, Chien-Han Hsiao, Chung-Cheng Yeh, Jian-Xun Lu, Chip-Jin Ng, Chieh-Ching Yen

**Affiliations:** 1 Department of Emergency Medicine, Chang Gung Memorial Hospital, Linkou Branch, Taoyuan 33305, Taiwan; yeh7504@gmail.com (H.-T.Y.); djf4siuol@hotmail.com (J.-X.L.); ngowl@ms3.hinet.net (C.-J.N.); 2 Department of Nephrology, Kidney Research Center, Chang Gung Memorial Hospital, Linkou Branch, College of Medicine, Chang Gung University, Taoyuan 33305, Taiwan; stevenliau0320@gmail.com; 3 Department of Emergency Medicine, Chang Gung Memorial Hospital, Keelung Branch, Keelung 20401, Taiwan; peidra.niu@gmail.com (K.-Y.N.); virous1128@gmail.com (C.-C.Y.); 4 Department of Linguistics, Indiana University, Bloomington, IN 47405, USA; chiehsia@indiana.edu; 5 Institute of Emergency and Critical Care Medicine, National Yang Ming Chiao Tung University, Taipei 11221, Taiwan

**Keywords:** dialysis, septic arthritis, length of stay

## Abstract

*Background and Objectives*: Septic arthritis is a medical emergency associated with high morbidity and mortality. The incidence rate of septic arthritis among dialysis patients is higher than the general population, and dialysis patients with bacteremia frequently experience adverse outcomes. The aim of this study was to identify the clinical features and risk factors for longer hospital length of stay (LOS), positive blood culture, and in-hospital mortality in dialysis patients with septic arthritis. *Materials and Methods*: The medical records of 52 septic arthritis dialysis patients admitted to our hospital from 1 January 2009 to 31 December 2020 were analyzed. The primary outcomes were bacteremia and in-hospital mortality. Variables were compared, and risk factors were evaluated using linear and logistic regression models. *Results*: Twelve (23.1%) patients had positive blood cultures. A tunneled cuffed catheter for dialysis access was used in eight (15.4%) patients, and its usage rate was significantly higher in patients with positive blood culture than in those with negative blood culture (41.7 vs. 7.5%, *p* = 0.011). Fever was present in 15 (28.8%) patients, and was significantly more frequent in patients with positive blood culture (58.3 vs. 20%, *p* = 0.025). The most frequently involved site was the hip (*n* = 21, 40.4%). The most common causative pathogen was Gram-positive cocci, with MRSA (*n* = 7, 58.3%) being dominant. The mean LOS was 29.9 ± 25.1 days. The tunneled cuffed catheter was a significant predictor of longer LOS (Coef = 0.49; Cl 0.25–0.74; *p* < 0.001). The predictors of positive blood culture were fever (OR = 4.91; Cl 1.10–21.83; *p* = 0.037) and tunneled cuffed catheter (OR = 7.60; Cl 1.31–44.02; *p* = 0.024). The predictor of mortality was tunneled cuffed catheter (OR = 14.33; Cl 1.12–183.18; *p* = 0.041). *Conclusions*: In the dialysis population, patients with tunneled cuffed catheter for dialysis access had a significantly longer hospital LOS. Tunneled cuffed catheter and fever were independent predictors of positive blood culture, and tunneled cuffed catheter was the predictor of in-hospital mortality. The recognition of the associated factors allows for risk stratification and determination of the optimal treatment plan in dialysis patients with septic arthritis.

## 1. Introduction

Septic arthritis is a medical emergency requiring timely antibiotic treatment and surgical intervention, and is associated with high morbidity and mortality due to cartilaginous breakdown and irreversible joint damage typically occurring within 48 h [1]. Septic arthritis frequently presents as a swollen, warm, extremely tender joint accompanied by systemic signs of infection, including fever and leukocytosis [2]. In the absence of trauma or recent instrumentation to the joint, septic arthritis is typically caused by hematogenous seeding. Common locations for septic arthritis include shoulders, hips, and knee joints, all involving metaphyses with ample blood supply, which leads to the proclivity for bacterial infection [3]. The most frequently identified causative organism in septic arthritis is *Staphylococcus aureus* (*S. aureus*), followed by methicillin-resistant *S. aureus* (MRSA), Group B *Streptococcus*, and Gram-negative organisms [4,5]. The risk factors for the development of septic arthritis include an age greater than 80 years, diabetes mellitus, rheumatoid arthritis, recent joint surgery, hip or knee prosthesis and/or skin infection, and human immunodeficiency virus type-1 infection as suggested by meta-analysis research [6].

The incidence rate of septic arthritis among dialysis patients has been shown to be 50 times higher than that of the general population [7], and the occurrence of this condition in dialysis patients frequently results in adverse outcomes, such as osteomyelitis, joint replacement, amputation, and mortality [8,9]. The elevated incidence of septic arthritis in dialysis patients is likely to be the consequence of compromised cellular and humoral immunity and frequent exposure to urinary and vascular access, which increases the risk of bloodstream infection [9]. Compared to the general population, dialysis patients with bacteremia are susceptible to higher morbidity and mortality [10]. However, the prognostic factors for in-hospital outcomes in dialysis patients with septic arthritis have yet to be identified in the current literature. The aim of this study was to evaluate the clinical characteristics and identify the predictors of hospital length of stay (LOS), positive blood culture, and in-hospital mortality in dialysis patients with septic arthritis.

## 2. Materials and Methods

### 2.1. Study Design and Setting

The present study was approved by the Chang Gung Medical Foundation Institutional Review Board (IRB no. 202102087B0, Date of Approval: 15 December 2021), and was conducted in accordance with the Declaration of Helsinki. The data were from a tertiary referral center with the capacity of 3700 beds, 100,000 annual admissions, and 200,000 annual emergency department (ED) visits in Taiwan. All adult patients who met the inclusion criteria of the study during the period of 1 January 2009, to 31 December 2020, were retrospectively enrolled for analysis.

### 2.2. Patient Selection and Data Collection

**Figure 1:**
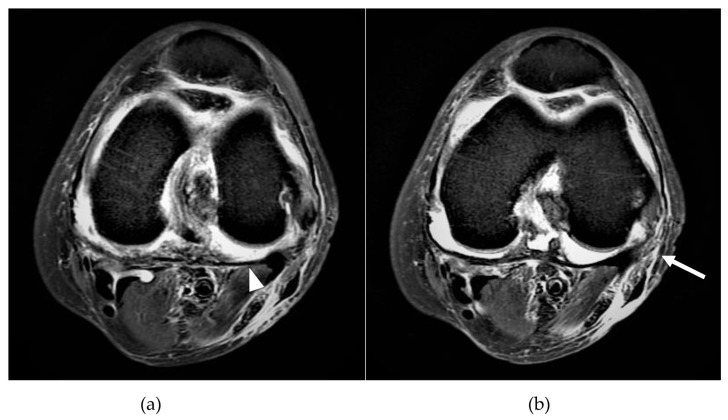
A 48-year-old male on regular hemodialysis presented to the emergency department with fever, left knee pain and swelling. The patient underwent T2-weighted fat-suppressed magnetic resonance imaging: (**a**) The homogenous signal in the left knee articular space was compatible with synovial effusion (arrowhead). (**b**) The synovial thickening and proliferation were consistent with inflammation (arrow).

By searching the electronic medical records (EMRs) during the study period, all the chronic dialysis patients diagnosed with septic arthritis at our hospital were first identified. A diagnosis of septic arthritis was made if the patient showed the clinical symptoms of septic arthritis and met the following criteria: (1) One or more positive culture or gram stain on joint aspiration or surgical specimens was detected. (2) Radiological findings—including evidence from radiographs, computed tomography (CT), magnetic resonance imaging (MRI), or bone scan—were consistent with septic arthritis (Figure 1). (3) Clinical response was present after the administration of effective antibiotics. Patients with an age under 18, incomplete medical records, or duplicated data were excluded. The patients selected by the EMRs were further reviewed by two physicians (H.-T.Y. and C.-C.Y.) for their inclusion eligibility.

For each identified patient, demographic information, which included age, sex, initial vital signs upon admission, and comorbidities such as hypertension, diabetes mellitus, heart failure (including heart failure with preserved or reduced ejection fraction), malignancy, gout, rheumatic arthritis, osteoarthritis, prosthetic joint, liver cirrhosis, and steroid use, were retrieved. Information regarding initial presentations, dialysis access, site of involved joint, laboratory findings, organisms identified from blood or synovial cultures, treatment modalities, and length of hospital stay was collected. Laboratory findings were obtained; of the 52 patients, the blood test results of white blood count (WBC) were available in 48 patients, hemoglobin in 48, platelet count in 46, international normalized ratio in 18, C-reactive protein (CRP) in 41, and culture in 52. The results of synovial fluid analyses that covered white blood count, percentage of polymorphonuclear cells, and culture were obtained for 32 patients.

The primary outcome was hospital LOS, and the secondary outcomes were positive blood culture and in-hospital mortality. Patients were followed up for one year for the identification of possible complications, including recurrence, joint replacement, amputation, osteomyelitis, and abscess formation. These complications were determined by reviewing imaging findings and surgical records in the EMRs under both outpatient and inpatient settings.

### 2.3. Statistical Analysis

Patient characteristics, comorbidities, initial presentations, dialysis access, site of involved joint, laboratory findings, organisms, treatment modalities, and outcomes were reported as numbers (percentages) for categorical variables and mean ± standard deviation (SD) for continuous variables. For the categorical variables, the characteristics of the bacteremia and non-bacteremia patients were compared using the Chi-square test or Fisher’s exact test as appropriate. For the continuous variables, independent Student’s *t*-tests were used for normally distributed variables, and Mann–Whitney U-tests were used for skewed variables. A linear regression model was used to determine the predictors of longer LOS. To identify the predictors of positive blood culture and in-hospital mortality, a univariate logistic regression was first performed, and the statistically significant risk factors (*p* < 0.05) were then selected and used in a multivariate logistic regression model. All analyses were performed using SPSS software v26.0 (SPSS Inc., Chicago, IL, USA). A two-sided *p* value of <0.05 was considered statistically significant.

## 3. Results

### 3.1. Patient Characteristics

**Figure 2:**
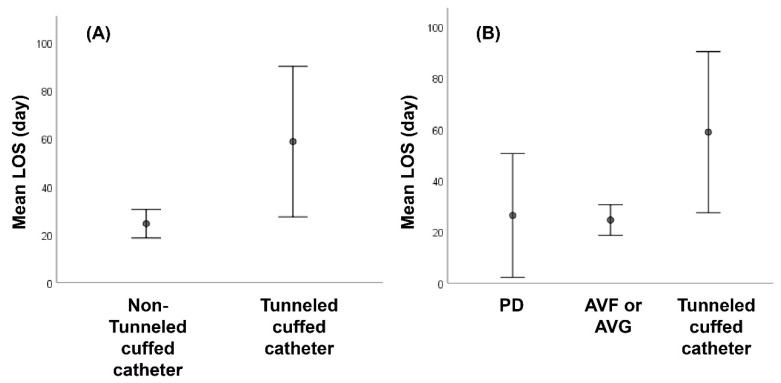
Comparison of mean LOS in dialysis patients with septic arthritis. (**A**) Stratified by dialysis access with or without tunneled cuffed catheter (58.8 ± 37.6 vs. 24.7 ± 18.3 days, *p* = 0.038) (**B**) Stratified by dialysis access with PD, AVF or AVG, and tunneled cuffed catheter. There were significant differences in LOS between patients with tunneled cuffed catheter and AVF or AVG (58.8 ± 37.6 vs. 24.5 ± 18.9 days, *p* = 0.001). There was no significant difference in LOS between patients with tunneled cuffed catheter and PD (58.8 ± 37.6 vs. 26.3 ± 9.7 days, *p* = 0.091), and PD and AVF or AVG (26.3 ± 9.7 vs. 24.5 ± 18.9 days, *p* = 0.990). LOS: length of stay; PD: peritoneal dialysis; AVF: arteriovenous fistula; AVG: arteriovenous graft.

A total of 52 patients met the inclusion criteria of the study. Their ages ranged from 28 to 88 years with a mean of 60.6 ± 12.9 years. The percentage of male patients was 46.2%. The mean LOS was 29.9 ± 25.1 days. A tunneled cuffed catheter for dialysis access was used in eight (15.4%) patients, peritoneal dialysis in three (5.8%) patients, and arteriovenous fistula and arteriovenous graft in 41 (78.8%) patients. Twelve (23.1%) patients had positive blood cultures. Fever was present in 15 (28.8%) patients and significantly more frequent in patients with positive blood culture (58.3% vs. 20%, *p* = 0.025). Of all the septic arthritis patients, 36 (69.2%) had hypertension, 29 (55.8%) had diabetes mellitus, 13 (25%) had heart failure, 11 (21.2%) had liver cirrhosis, 10 (19.2%) had prosthetic joints, 5 (9.6%) had osteoarthritis, 4 (7.7%) had gout, 3 (5.8%) had malignancy, 2 (3.8%) had rheumatic arthritis, and 2 (3.8%) had steroid use. All patients manifested as acute monoarthritis, and the most frequently involved sites were the hip (*n* = 21, 40.4%), followed by the knee (*n* = 18, 34.6%), the shoulder (*n* = 9, 17.3%), the ankle (*n* = 3, 5.8%), and the elbow (*n* = 1, 1.9%). All patients presented with joint pain, with joint swelling reported in 22 (42.3%) patients, joint redness in 16 (30.8%), and joint with a limited range of motion in 22 (42.3%). For the laboratory findings, CRP levels were elevated (>5 mg/L) in all the patients (mean (range): 160.5 (20–463) mg/L), and leukocytosis (WBC > 11,000/uL) was present in 24 (46.2%) patients (mean (range): 12,237 (3400–30,600) µL). When patients were divided into tunneled cuffed catheter group and non-tunneled cuffed catheter group, the mean LOS was significantly longer (58.8 ± 37.6 vs. 24.7 ± 18.3 days, *p* = 0.038) (Figure 2) and the proportion of positive blood culture was significantly higher (62.5% vs. 15.9%, *p* = 0.004) in patients with tunneled cuffed catheter (Table 1).

### 3.2. Microbiology Results

Blood culture was performed in all 52 patients to identify the causative pathogen, and 12 (23.1%) patients showed evidence of bacteremia. The most common pathogens were Gram-positive cocci, including MRSA (*n* = 7, 58.3%), methicillin-susceptible *S. aureus* (MSSA) (*n* = 2, 16.7%), and Group B Streptococcus (*n* = 1, 8.3%), followed by Gram-negative bacilli, including Salmonella Group D (*n* = 1, 8.3%) and Escherichia coli (*n* = 1, 8.3%). Synovial fluid culture was obtained in 32 patients, and the results were positive in 21 (65.6%) of them. MRSA was the causative pathogen in 10 (47.6%) patients, MSSA in 2 (9.5%), Methicillin-susceptible Staphylococcus lugdunensis in 1 (4.8%), Staphylococcus hominis in 1 (4.8%), Group B Streptococcus in 2 (9.5%), Vancomycin-resistant Enterococcus in 1 (4.8%), Enterococcus faecalis in 1 (4.8%), Salmonella group D in 1 (4.8%), Escherichia coli in 1 (4.8%), and Candida famata in 1 (4.8%) (Table 2).

### 3.3. Treatment and Outcomes

Each patient received intravenous antibiotics once septic arthritis was diagnosed. In total, 26 (50%) patients required surgical debridement, with the percentage being significantly lower in patients with positive blood culture than in those with negative blood culture (25% vs. 57.5%, *p* = 0.048). There were no significant differences in mean LOS across the patient groups with different age, sex, vital signs, comorbidities, initial presentations, sites of involved joints, and reception of surgical debridement. Five (9.6%) patients were admitted to the intensive care unit (ICU), and three (5.8%) died (Table 3). In regard to one-year complications, 8 (15.4%) patients had recurrent septic arthritis, 15 (28.8%) had joint replacement, 2 (3.8%) had amputation, 1 (1.9%) had osteomyelitis, and 1 (1.9%) had local abscess formation (Table 4).

### 3.4. Univariate and Multivariate Analyses of Predictors of Longer LOS, Positive Blood Culture, and In-Hospital Mortality

To determine the predictors of longer LOS, a univariate linear regression model was used, and the model demonstrated that tunneled cuffed catheter was the only statistically significant predictor (Coef = 0.49; Cl 0.25–0.74; *p* < 0.001) (Table 5). The multivariate regression analysis was thereby not performed. To identify the predictors of positive blood culture and in-hospital mortality, we employed a univariate logistic regression model followed by a multivariate logistic regression model. For positive blood culture, the univariate logistic regression identified fever (OR = 5.60; Cl 1.40–22.36; *p* = 0.015) and tunneled cuffed catheter (OR = 8.81; Cl 1.70–45.58; *p* = 0.009) as statistically significant predictors. In the multivariate regression model, fever (OR = 4.91; Cl 1.10–21.83; *p* = 0.037) and tunneled cuffed catheter (OR = 7.60; Cl 1.31–44.02; *p* = 0.024) (Table 6) remained statistically significant predictors of positive blood culture. For in-hospital mortality, the univariate logistic regression identified a single statistically significant predictor of tunneled cuffed catheter (OR = 14.33; Cl 1.12–183.18; *p* = 0.041), and multivariate regression analysis was thereby not performed (Table 7).

## 4. Discussion

This retrospective study provides a documentation of the clinical characteristics in dialysis patients with septic arthritis, and analyzes the predictors of in-hospital outcomes—LOS, positive blood culture, and in-hospital mortality—which have not been previously reported. Our results showed that patients with tunneled cuffed catheter had significantly longer LOS. The independent predictors of positive blood culture were tunneled cuffed catheter for dialysis access and fever at the triage, and the independent predictor of in-hospital mortality was tunneled cuffed catheter.

With an incidence rate of 500 cases per 100,000 patients every year in the U.S. [8], dialysis patients have been reported to be more likely to develop septic arthritis than the general population due to their susceptibility to bacteremia [11]. Studies have shown that vascular access for dialysis is a major risk factor for bacteremia in patients with dialysis [12,13]. As reflected in our study, tunneled cuffed catheter was an independent risk factor for longer LOS, positive blood culture, and in-hospital mortality in septic arthritis dialysis patients. The general incidence rate of tunneled catheter-related bacteremia has been reported to be 3 per 1000 catheter days [14]. Impaired immunity, comorbidities, malnutrition—which enhances the virulence properties of hospital bacteria—as well as the breakdown of the protective anatomical barriers due to repeated intravascular intervention are the main reasons for the prevalence of bloodstream infection in these patients. Infection complications—which include endocarditis, vertebral osteomyelitis or discitis, and less commonly, spinal epidural abscess, septic arthritis, and septic pulmonary emboli—occur in 15–40% of catheter-related bloodstream infections [15,16].

While studies have shown that old age, heart failure, diabetes mellitus, and surgical treatment are associated with longer hospital LOS in patients with septic arthritis [17,18], there has not been a systematic analysis of the risk factors for longer LOS in the dialysis population. To our knowledge, the current study is the first to report tunneled cuffed catheter as a significant risk factor for longer LOS in dialysis patients. The tunneled cuffed catheters may serve as a route of bacteria entrance and, in turn, lead to an increase in patients’ risk for developing bacteremia and further contribute to high morbidity and mortality [12,13]. With longer LOS, patients with tunneled cuffed catheter may also undergo a longer period of antibiotic therapy and management of potential complications, which implies greater healthcare resource burden for the hospital and higher medical expenses for patients. The recognition of these associated factors is crucial to risk stratification and determination of the optimal treatment plan for each patient.

Septic arthritis is an orthopedic emergency for which delays in treatment can result in joint degradation, osteonecrosis, and instability [19]. The treatment for this condition requires careful evaluation of the need for surgical intervention as previous studies have suggested that medical treatment may be as effective as surgical treatment, and is associated with shorter hospital stay and better functional outcomes [20,21,22]. As for surgical intervention, the optimal surgical approach to septic arthritis is best determined by an orthopedic surgeon based on the involved joint and associated clinical factors. In the present study, a lower proportion of surgical debridement interventions was seen in patients with bacteremia than those without bacteremia. This may reflect the physicians’ attempt to avoid postoperative complications as an elevated postoperative complication rate has been reported in bacteremia patients; a prospective observational study conducted by Masaya et al. demonstrated that the incidence rate of any surgical site infection was significantly higher in patients with positive preoperative blood culture than in other patients [23].

Fever is the most frequent manifestation that elicits the suspicion of blood stream infection, and it was associated with a significantly higher risk of positive blood culture in our septic arthritis dialysis patients [24,25]. However, Sarah et al. observed that despite concurrent *S. aureus* bacteremia, over half of the dialysis patients did not have a detectable fever (temperature > 100.4 °F) when initially assessed at ED triage [26]. One possible reason is that dialysis patients tend to have low basal body temperatures, and resultantly, their maximal temperatures during an active infection may be lower than those of the normal population [27]. Consistent with the prior report, our study showed that fever occurred in only 28.8% of dialysis patients with septic arthritis, and in 58.3% of those with positive blood culture. Since dialysis patients with bacteremia have been found to have a blunted febrile response, routine testing for blood culture in dialysis patients with septic arthritis regardless of body temperature on initial presentation is recommended.

The causative organisms in this study were predominantly Gram-positive bacteria, with *S. aureus* and Streptococci attributable for more than 80% of these cases. MRSA was accountable for septic arthritis in more than half of the patients with bacteremia (58.3%) and in almost half of the patients who had positive synovial fluid cultures (47.6%)—both of these prevalence rates were higher than that of the general population [4,28,29]. The incidence rate of MRSA infection in dialysis patients in this study is noticeably higher than the rates reported in septic arthritis studies with or without dialysis status [9,29,30]. A higher risk of MRSA bacteremia in dialysis patients along with increased incidence of MRSA infections in both healthcare and community settings may explain this difference [31,32,33]. According to a Centers for Disease Control and Prevention report of the United States in 2005, invasive MRSA infections occurred in 45.2/1000 dialysis patients, a rate more than 100 times higher than that in non-dialysis patients. In a single-center surveillance report of Brazil from 2005–2008, a total of 3907 Gram-positive cocci were analyzed, and 31.0% of *S. aureus* was MRSA strains, which had an increased incidence rate of 38.5% from 2010–2013 [34,35]. Another study analyzing data from the National Inpatient Sample revealed that MRSA-related septicemia hospitalizations increased from 1.67 to 1.94 discharges per 1000 hospitalizations between 2016 and 2019 [36]. Dialysis patients are highly susceptible to the colonization and infection of MRSA due to immunosuppression, repeat hospitalization, and the frequent use of antibiotics. Exposure to invasive procedures and regular contact with other colonized patients and healthcare workers are also considerable risk factors [37]. Because of its high incidence, the timely initiation of empirical antimicrobial therapy with MRSA coverage is necessary for dialysis patients with septic arthritis.

The in-hospital mortality rate of septic arthritis in dialysis patients was 5.76% in our study. In the literature, the mortality rate of septic arthritis ranges from 4% to 42% [38]. This broad range may be due to study-to-study differences in the patients’ comorbidities, inclusion criteria (children, prosthetic joint infections, etc.), and clinical presentations. As for the general population, the mortality rate of septic arthritis was reported to be 5.6%, 7.7%, and 5.5% in Ferrand et al., Munoz-Egea et al., and Lim et al., respectively [39,40,41]. Our findings revealed that the mortality in dialysis patients with septic arthritis at our institution is comparable to that of the general population. We hypothesize that appropriate evaluation and intervention by a multidisciplinary team may improve treatment outcomes in dialysis patients with septic arthritis.

Despite our findings, this study has several limitations. First, due to its retrospective nature, this study was inevitably limited by missing data and did not allow for uniform controlled collection of the clinical variables; for example, synovial fluid analysis was not performed in all the patients. Second, because of the rarity of the disease, our sample size was small and may have limited the statistical power and generalizability of our findings. Third, being a single center study, our findings may not extend to the broader population. Future prospective multicenter studies may be necessary to further verify our findings.

## 5. Conclusions

Septic arthritis is a medical emergency requiring early recognition and timely treatment to minimize joint damage, and reduce associated morbidity and mortality. In the dialysis population, patients with tunneled cuffed catheter had a significantly longer LOS. Tunneled cuffed catheter for dialysis access and fever were independent predictors of positive blood culture, and tunneled cuffed catheter was the predictor of in-hospital mortality. The recognition of these associated factors allows for risk stratification and determination of the optimal treatment plan in dialysis patients with septic arthritis.

## Figures and Tables

**Table 1:** Demographics, clinical characteristics, and laboratory results of patients with and without tunneled cuffed catheter.

Variable	Total (*n* = 52)	Tunneled Cuffed Catheter (*n* = 8)	Non-Tunneled Cuffed Catheter (*n* = 44)	*p* Value
Age (year)	60.6 ± 12.9	61.9 ± 13.6	60.4 ± 13.0	0.768
Male	24 (46.2)	3 (37.5)	21 (47.7)	0.711
Systolic blood pressure (mmHg)	137.4 ± 40.8	117 ± 38.6	141.5 ± 40.4	0.122
Diastolic blood pressure (mmHg)	74.2 ± 22.6	69.3 ± 15.2	75.2 ± 23.9	0.501
Heart rate (beats/min)	98.5 ± 15.3	102.5 ± 18.7	97.7 ± 14.7	0.422
Fever ^†^	15 (28.8)	4 (50.0)	11 (25.0)	0.207
Initial presentations				
Joint pain	52 (100)	8 (100)	44 (100)	
Joint swelling	22 (42.3)	1 (12.5)	21 (47.7)	0.118
Joint redness	16 (30.8)	1 (12.5)	15 (34.1)	0.409
Limited range of motion	22 (42.3)	1 (12.5)	21 (47.7)	0.118
Joint involved				0.823
Hip	21 (40.4)	4 (50.0)	17 (38.6)	
Knee	18 (34.6)	2 (25.0)	16 (36.4)	
Shoulder	9 (17.3)	1 (12.5)	8 (18.2)	
Ankle	3 (5.8)	1 (12.5)	2 (4.5)	
Elbow	1 (1.9)	0 (0.0)	1 (2.3)	
Comorbidities				
Hypertension	36 (69.2)	3 (37.5)	33 (75.0)	0.089
Diabetes mellitus	29 (55.8)	2 (25.0)	27 (61.4)	0.118
CAD	13 (25.0)	1 (12.5)	12 (27.3)	0.662
HFpEF or HFrEF	13 (25.0)	2 (25.0)	11 (25.0)	1.000
Malignancy	3 (5.8)	0 (0.0)	3 (6.8)	1.000
Gout	4 (7.7)	0 (0.0)	4 (9.1)	1.000
Rheumatic arthritis	2 (3.8)	0 (0.0)	2 (4.5)	1.000
Osteoarthritis	5 (9.6)	0 (0.0)	5 (11.4)	1.000
Prosthetic joint	10 (19.2)	0 (0.0)	10 (22.7)	0.328
Liver cirrhosis	11 (21.2)	2 (25.0)	9 (20.5)	1.000
Steroid use	2 (3.8)	0 (0.0)	2 (4.5)	1.000
Blood test ^†^				
White blood count (10^3^/µL) *n* = 48	12.2 ± 5.8	14.6 ± 7.8	11.7 ± 5.3	0.168
Leukocytosis ^‡^	24 (50.0)	6 (75.0)	18 (45.0)	0.245
Hemoglobin (g/dL) *n* = 48	9.5 ± 2.1	9.9 ± 2.2	9.4 ± 2.1	0.552
Platelet (10^3^/µL) *n* = 46	236.7 ± 120.4	264.6 ± 141.2	230.8 ± 116.9	0.477
INR *n* = 18	1.2 ± 0.4	1.2 ± 0.0	1.2 ± 0.4	0.956
C-reactive protein (mg/L) *n* = 41	160.5 ± 98.6	194.8 ± 100.0	152.2 ± 97.9	0.278
Positive culture	12 (23.1)	5 (62.5)	7 (15.9)	0.004 *
Synovial fluid analyses ^§^				
White blood count (10^3^/mm^3^)	69.2 ± 99.1	39.6 ± 43.6	79.1 ± 111.2	0.454
PMN (%)	85.2 ± 16.5	92.2 ± 5.4	83.2 ± 18.1	0.292
Positive culture	21 (65.6)	2 (40.0)	19 (70.4)	0.189

Count data are expressed as number (percentage), and continuous values are expressed as mean ± SD. AVF: Arteriovenous fistula; AVG: Arteriovenous graft; HFpEF: Heart failure with preserved ejection fraction; HFrEF: Heart failure with reduced ejection fraction; COPD: Chronic obstructive pulmonary disease; CAD: Coronary artery disease; INR: International normalized ratio; PMN: Polymorphonuclear leukocytes. ^†^ Defined as body temperature > 37.7 °C. ^‡^ Leukocytosis was defined as white blood count > 11,000/µL, and blood culture data were available in all patients. ^§^ Synovial fluid culture data were available in 32 patients, and routine data in 21 patients. * *p* value < 0.05.

**Table 2:** Microbiology results of blood cultures and synovial fluid cultures.

Microorganism	*n*	%
Blood cultures (*n* = 12)		
Methicillin-resistant Staphylococcus aureus	7	58.3
Methicillin-susceptible Staphylococcus aureus	2	16.7
Group B Streptococcus	1	8.3
Salmonella group D	1	8.3
Escherichia coli	1	8.3
Synovial fluid cultures (*n* = 21)		
Methicillin-resistant Staphylococcus aureus	10	47.6
Oxacillin-susceptible Staphylococcus aureus	2	9.5
Oxacillin-susceptible Staphylococcus lugdunensis	1	4.8
Staphylococcus hominis	1	4.8
Group B Streptococcus	2	9.5
Salmonella group D	1	4.8
Escherichia coli	1	4.8
Vancomycin-resistant Enterococcus	1	4.8
Enterococcus faecalis	1	4.8
Candida famata	1	4.8

**Table 3:** Patients with ICU admission.

Case#	Age	Gender	Past Medical History	Joint	Dialysis Route	Surgical Debridement	Antibiotic Therapy	Length of Stay (Days)	In-Hospital Mortality
1	48	Female	HFpEF, HTN	Knee	Tunneled cuffed catheter	Y	D1-D7: Oxacillin, Ceftriaxone D8-D20: Ceftazidime, Vancomycin D21-D26: Imipenem/Cilastatin, Teicoplanin, Micafungin	28	Y
2	67	Male	DM, HTN, liver cirrhosis	Hip	AVG	N	D1-D13: Oxacillin	12	Y
3	85	Male	DM	Ankle	Tunneled cuffed catheter	N	D1-D5: Ertapenem, Vancomycin D6-D15: Daptomycin	28	Y
4	60	Male	DM, HTN, osteoarthritis	Hip	AVG	Y	D1-D7: Vancomycin	13	N
5	73	Male	HTN, HF_P_EF, DM, CAD	Hip	AVF	Y	D1-D2: Oxacillin D3-D4: Gentamycin D5-D12: Teicoplanin, Ceftriaxone D13-D30: Cotrimoxazole	12	N

HFpEF: heart failure with preserved ejection fraction; HTN: hypertension; DM: diabetes mellitus; CAD: coronary artery disease; AVG: arteriovenous graft; AVF: arteriovenous fistula; Y: yes; N: no; D: day.

**Table 4:** Comparison of treatment, in-hospital outcomes, and 1-year complications in patients with and without tunneled cuffed catheter.

Variable	Total *n* = 52	Tunneled Cuffed Catheter (*n* = 8)	Non-Tunneled Cuffed Catheter (*n* = 44)	*p* Value
Treatment				
Surgical debridement	26 (50)	4 (50.0)	22 (50.0)	1.000
Duration of antibiotic treatment (days) ^†^	29.6 ± 26.8	57.1 ± 45.0	24.9 ± 19.7	0.108
In-hospital outcomes				
Length of stay	29.9 ± 25.1	58.8 ± 37.6	24.7 ± 18.3	0.038 *
Intensive care unit admission	5 (9.6)	2 (25.0)	3 (6.8)	0.164
Death	3 (5.8)	2 (25.0)	1 (2.3)	0.011 *
1-year complications				
Recurrence	8 (15.4)	1 (12.5)	7 (15.9)	1.000
Joint replacement	15 (28.8)	1 (12.5)	14 (31.8)	0.412
Amputation	2 (3.8)	0 (0.0)	2 (4.5)	1.000
Osteomyelitis	1 (1.9)	0 (0.0)	1 (2.3)	1.000
Abscess formation	1 (1.9)	0 (0.0)	1 (2.3)	1.000

Count data are expressed as number (percentage), and continuous values are expressed as mean ± SD. ^†^ Including inpatient and outpatient treatment. * *p* value < 0.05.

**Table 5:** Univariate analyses of predictors of longer LOS in dialysis patients with septic arthritis using the linear regression model.

	Univariate	
	Coef (95%CI)	*p* Value
Age	−0.09 (−0.29, 0.14)	0.408
Male	−0.09 (−0.37, 0.20)	0.536
Fever	0.07 (−0.22, 0.35)	0.644
Joint redness	−0.08 (−0.36, 0.21)	0.592
Tunneled cuffed catheter	0.49 (0.25, 0.74)	<0.001 *
Diabetes mellitus	−0.26 (−0.53, 0.02)	0.068
Liver cirrhosis	0.12 (−0.16, 0.40)	0.385
Congestive heart failure	0.09 (−0.19, 0.37)	0.527
Gout	−0.02 (−0.30, 0.27)	0.893
Rheumatic arthritis	−0.14 (−0.42, 0.14)	0.323
Osteoarthritis	−0.06 (−0.35, 0.22)	0.664
Prosthetic joint	−0.13 (−0.41, 0.15)	0.360
Surgical debridement	0.03 (−0.25, 0.32)	0.824
Leukocytosis	0.25 (−0.05, 0.51)	0.098

Coef: Coefficient; 95% CI: 95% confidence interval. * *p* value < 0.05.

**Table 6:** Univariate and multivariate analyses of predictors of positive blood culture in dialysis patients with septic arthritis using the logistic regression model.

	Univariate		Multivariate	
	OR (95%CI)	*p* Value	OR (95%CI)	*p* Value
Age	1.00 (0.95,1.05)	0.850		
Male	0.79 (0.21,2.91)	0.723		
Fever	5.60 (1.40,22.36)	0.015	4.91 (1.10,21.83)	0.037 *
Joint redness	1.88 (0.49,7.20)	0.355		
Tunneled cuffed catheter	8.81 (1.70,45.58)	0.009	7.60 (1.31,44.02)	0.024 *
Diabetes mellitus	0.74 (0.20,2.70)	0.647		
Liver cirrhosis	0.69 (0.13,3.73)	0.666		
Congestive heart failure	1.00 (0.23,4.44)	1.000		
Gout	0.64 (0.07,6.05)	0.694		
Rheumatic arthritis	3.55 (0.21,61.38)	0.384		
Osteoarthritis	2.47 (0.36,16.84)	0.357		
Prosthetic joint	0.80 (0.15,4.40)	0.797		
Leukocytosis	4.20 (0.97,18.18)	0.055		

OR: odds ratio; 95% CI: 95% confidence interval. * *p* value < 0.05.

**Table 7:** Univariate analysis of predictors of in-hospital mortality in dialysis patients with septic arthritis using the logistic regression model.

	Univariate	
	OR (95%CI)	*p* Value
Age	1.04 (0.94,1.16)	0.403
Male	2.46 (0.21,28.89)	0.475
Fever	5.54 (0.46,66.32)	0.177
Joint redness	1.10 (0.09,13.09)	0.940
Tunneled cuffed catheter	14.33 (1.12,183.18)	0.041 *
Diabetes mellitus	1.63 (0.14,19.18)	0.698
Liver cirrhosis	1.95 (0.16,23.73)	0.600
Congestive heart failure	1.54 (0.13,18.54)	0.733
Gout	5.63 (0.41,76.43)	0.194
Rheumatic arthritis	7.68 (0.53,110.65)	0.135
Osteoarthritis	3.58 (0.28,45.80)	0.326
Prosthetic joint	2.22 (0.18,27.26)	0.532
Surgical debridement	0.48 (0.04,5.65)	0.559
Leukocytosis	2.09 (0.18,1.00)	0.502

OR: Odds ratio; 95% CI: 95% confidence interval. * *p* value < 0.05.

## Data Availability

Not applicable.
